# Q&A: modern crop breeding for future food security

**DOI:** 10.1186/s12915-019-0638-4

**Published:** 2019-02-25

**Authors:** Kai P. Voss-Fels, Andreas Stahl, Lee T. Hickey

**Affiliations:** 1 Queensland Alliance for Agriculture and Food Innovation, The University of Queensland, St Lucia, QLD 4072 Australia; 2 Department of Plant Breeding, IFZ Research Centre for Biosystems, Land Use and Nutrition, Justus Liebig University, Heinrich-Buff-Ring 26-32, 35392 Giessen, Germany

## Abstract

Farmers around the world have recently experienced significant crop losses due to severe heat and drought. Such extreme weather events and the need to feed a rapidly growing population have raised concerns for global food security. While plant breeding has been very successful and has delivered today’s highly productive crop varieties, the rate of genetic improvement must double to meet the projected future demands. Here we discuss basic principles and features of crop breeding and how modern technologies could efficiently be explored to boost crop improvement in the face of increasingly challenging production conditions.

## What is the demand for plant-based food products?

The current number of 7.6 billion people on this planet is estimated to increase to about 10 billion by 2050. With this rapid population growth the world has become increasingly urbanized and the ratio of food producers to food consumers has significantly declined. This has placed pressure on food production globally, but intensified, more efficient agricultural production has met these demands. There are, however, serious concerns that the forecasted increase in demand for plant-based products by up to 70% within the next three to four decades cannot be met through increased production using current crop varieties and farming practices. This represents an unprecedented challenge for all related fields of agricultural and environmental research.

## Which crops do we eat?

Although about 300,000 plant species are edible, only a fraction of them are used for human nutrition. Around 200 species are regularly consumed, and only three of them—rice, maize and wheat—provide 60% of the energy in the human diet. In addition to cereals, other major food crops include roots and tubers, sugars, pulses, nuts, oil-bearing crops, vegetables, fruits, spices, and others such as tea and coffee. While food crops are grown and harvested for profit in many developed countries, they make an important contribution to food security through global trade. In developing countries, subsistence farmers often grow food crops where hunger is still a serious issue. Many crops important for local diets in developing countries have received little investment, known as orphan crops (e.g. sorghum, finger millet and cassava), and with some rapid genetic improvement they could be cultivated more broadly to help diversify human diets and improve farming systems through better crop rotations.

## How did our major food crops evolve?

**Fig. 1.**
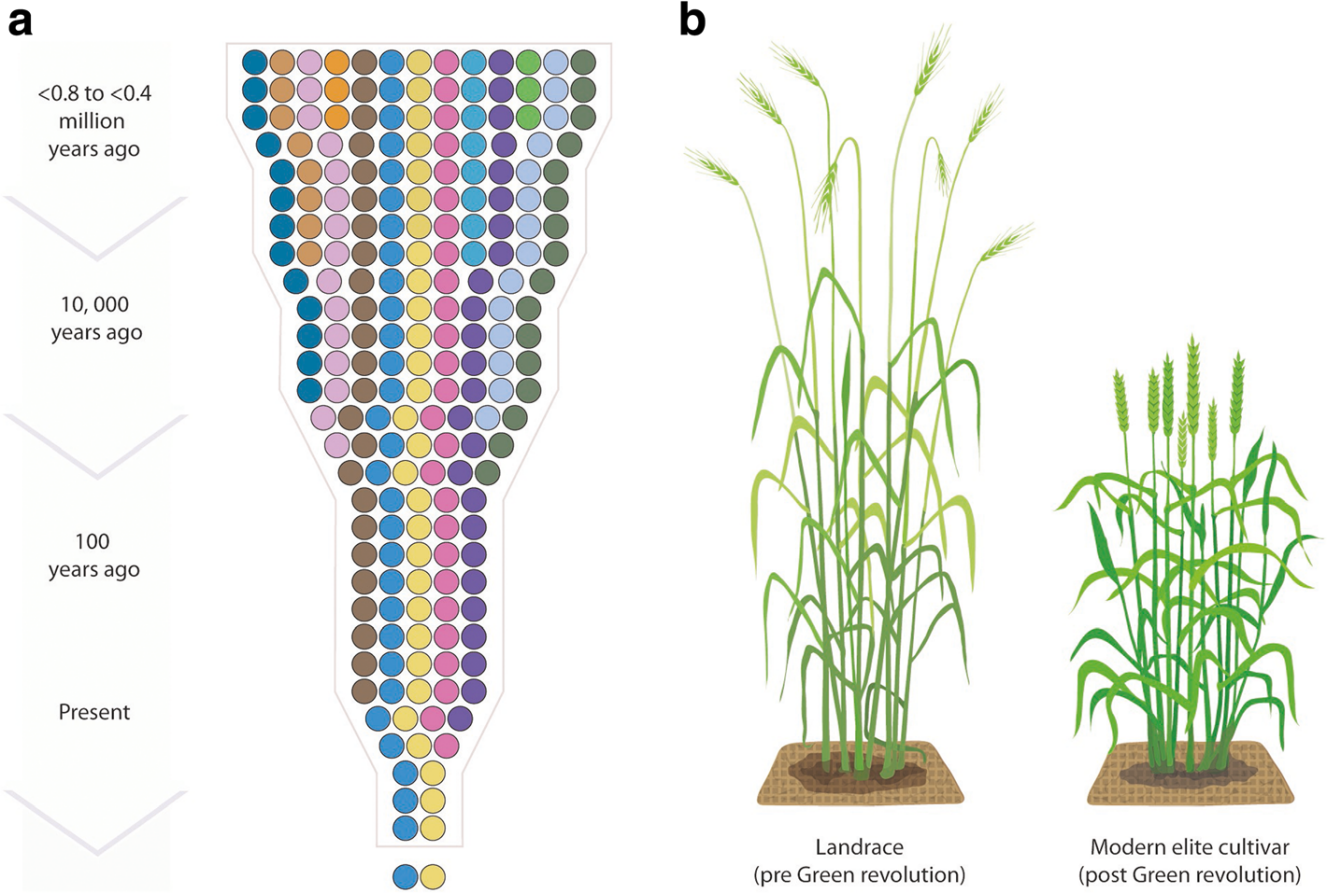
Development of crops over time, including **a** the loss of the diversity through the genetic bottlenecks of domestication, selection of landraces and modern plant breeding (adapted from [10], with permission from AAAS), and **b** example of a tall wheat landrace grown prior to the Green Revolution (*left*) and a modern high yielding cultivar selected for reduced plant height (*right*)

The beginning of crop development dates far back into human history and is widely accepted to have its origin in the ‘Fertile Crescent’, a region that today spans part of the countries of Iraq, Palestine, Syria, Lebanon, Cyprus, Jordan, Israel, Egypt, Turkey and Iran. Recently, new evidence emerged that prehistoric bread-like products were produced in South-west Asia 14,400 years ago [1], which were made from root tubers (*Bolboschoenus glaucus*) and seeds of wild einkorn (*Triticum boeoticum*), one of the ancestors of today’s wheat. Interestingly, this pushes back the evidence of bread to at least 4000 years before agriculture emerged. This suggests that early bread-making culture may have fuelled domestication of our first crops [1]. During settlement and the advent of agriculture, humans selected the most favourable plants of the available ancestral types, and this process of co-evolution between plant species and humankind resulted in today’s food crops. Seeds from the best performing plants were retained after harvest and sowed in the next season, leading to a continuous improvement of characteristics favourable for human nutrition and local production. This first form of breeding selection without any enforced crossing represented the main form of plant improvement for several thousands of years, shifting plant characteristics to increase their usefulness [2]. So-called domestication traits were the prerequisite for successful cultivation. A good example was the elimination of the seed dispersal mechanism in cereals like wheat and barley, known as seed shattering. While seed shattering at maturation is essential for wild grasses to disperse and reproduce, this characteristic is undesirable for farming. Therefore, plant genotypes that retained their seeds and thereby showed reduced yield losses were selected in the process of domestication. Because only few plants carried the desirable mutations, the strong selection pressure acted as a genetic bottleneck on the diversity available in our modern crops (Fig. 1a). Considering the extremely long evolution of crops, modern plant breeding has only recently been practiced, mainly after the formulation of Mendel’s Laws of Heredity in 1865. Mendel’s early genetic studies on peas and his resulting theories about inheritance and trait segregation paved the way for targeted crossing between parental genotypes, a practice that underpins modern crop improvement. However, in order to be able to meet the increasing demand for plant-based products, rates of genetic improvement must be doubled by the middle of this century.

## Why the need for speed?

New technology and advances in science offer new opportunities to further improve the efficiency of agriculture while reducing its environmental footprint, as well as enrich human diets with more nutritious foods. Since the Green Revolution, steady increases in crop productivity have occurred; however, there is concern that yield improvement is beginning to plateau. The current rate of annual yield improvement for major crops ranges between 0.8 and 1.2%, which must be doubled in order to meet the highly increased future demand for plant-based products [3]. Without new approaches that help boost productivity of staple crops through genetic improvement, global food security will be severely compromised in the next two to three decades, given the current global consumer behaviour. There are two main avenues by which crop productivity on farms can be improved: i) through the deployment of genetically superior crop varieties, or ii) via the adoption of better management practices. Ideally, both are addressed in parallel to provide a step-change in productivity, similar to what was achieved during the Green Revolution (see below). One of the major bottlenecks of plant breeding is the time it takes to develop an improved crop variety. Traditionally, it can take one or two decades because of the many steps of crossing, selection and testing required. Therefore, plant breeders and researchers around the world are developing new technologies and approaches to help speed up the efficiency of crop breeding. On farms, the adoption of poor or suboptimal management practices results in a yield ‘gap’, where the potential of crop yields are not realised. This gap exists even in developed countries, but is often largest in developing countries where machinery and other equipment and supplies, along with agronomy advice, are not readily available. Closing the yield gap is considered a challenging, yet high-priority, goal for enhancing productivity and global food security.

## What determines crop yield?

One of the most important traits that plant breeders aim to improve is ‘yield’. Depending on the species this can be ‘grain yield’, ‘total biomass’ or ‘total amount of sugar’ per area harvested. Yield represents a highly quantitative trait, which means that it is determined by numerous factors, including the interplay of many underlying genes with typically small effects (G), the environmental conditions under which the plants are grown (E) and the management practices applied (M). In most circumstances, there is a strong interaction among the G, E and M, which results in a high degree of complexity at the level of trait expression [4]. Each of those main complexes unfolds into several components that themselves represent complexes as well. In cereals, for example, the G component for grain yield can be understood as a higher-level complex consisting of several genetically determined components that jointly affect yield. For a crop like wheat, important yield components include the number and size of kernels per ear (e.g. per spike or panicle) and the number of ear-producing tillers per plant. Each of those major yield components unfolds into several lower-level physiological components, for example the number of spikelets per spike and the number of kernels that are produced per spikelet. The genetic constitution of a variety directly determines its yield potential, e.g. when considering genetic resistances against plant diseases caused by fungal or bacterial pathogens. Major environmental effects that are relevant to plant breeding are the amount of water that is available for the plant (mostly determined by frequency and distribution of precipitation), soil composition, radiation intensity and temperature. Interactions of G and E are most extreme when the order of merit of different varieties changes depending on the environmental conditions they were grown in. For example, a maize variety that produces a very shallow root system and allocates relatively limited resources to below-ground plant development might be able to produce significantly higher yields than a variety that produces a large, deep-reaching root system when both varieties are grown at a location characterised by sandy soils with a low water storage capacity and frequent rainfalls throughout the growing season. However, the situation changes entirely when both varieties are grown on production sites that are characterised by deep soils with a high water storage capacity and extremely low precipitation throughout the crop-growing season, leading to severe droughts. Especially phenology-related traits like flowering time can also play a key role in determining the plants’ performance in a given environment.

## How does plant breeding work?

The plant kingdom is extremely complex and the optimal plant breeding strategy is highly species-dependant. However, any breeding program can be broadly classed into three main processes: i) the creation of new genetic variation, ii) the selection of candidates based on defined merits and iii) the testing, propagation and release of improved crop varieties. The conventional way of creating new genetic variation is to make targeted crosses between selected individuals to create progeny that segregate for the trait of interest, typically representing the start of a breeding program. After that, a main task of the breeder is to identify genetically superior individuals from typically large populations (thousands to tens of thousands of genotypes). This typically involves multi-year and -location testing of candidates in replicated experimental field trials in order to estimate the genetic potential of a genotype across a range of growing conditions as accurately as possible. It is important to consider that most important crop species can be propagated as inbred lines or clones, thereby allowing repeated testing of the same genotype in different production conditions. For most important crop species, modern selection strategies have been developed that incorporate genome information based on next-generation DNA sequencing technologies in the breeding process (see below). In the final stage, breeders will typically register their most promising variety candidates (typically only one or two) at a legal variety testing department that runs multi-year and -location evaluation trials to assess if the variety has distinctly improved characteristics that warrant its official registration. Once registered, the new variety becomes available to farmers. Depending on the crop this process can take up to one to two decades, making breeding programs very rigid and complex endeavours.

## How does the mode of reproduction determine the breeding strategy?

The main principle ways of sexual propagation relevant to most crop species are outcrossing and inbreeding [4]. Outcrossing species sexually reproduce through hybridization of gametes from two different plants, whereas for inbreeding species both gametes originate from the same plant. Many important cereal crops, such as wheat and barley, are inbred species that produce hermaphrodite flowers. These flowers have biological mechanisms that minimise outcrossing. Classical breeding strategies that have been widely used for these crops are referred to as ‘pedigree breeding’ approaches, typically resulting in fully homozygous line varieties. Here, plant breeders make crosses by manually removing anthers from the ‘female’ plant (known as emasculation) and then apply pollen from a different ‘male’ plant. In this way, directed crosses can be made even for inbreeding species. Depending on the size of the breeding program the total number of directed crosses can range between less than 100 to a few thousand. The offspring segregate and breeders select the best plants during multiple rounds of recurrent inbreeding and field testing. A high level of homozygosity is critical to ensure that the variety that is grown by farmers does not segregate further, potentially exposing recessive genetic variants with detrimental effects on agronomically important traits. In outcrossing species, breeders aim to improve a plant population from which the best individuals are recurrently selected and intercrossed during the breeding program, making it conceptually different from line breeding for inbreds, which results in one single, improved genetically homozygous line. The rate of success of population breeding programs depends on whether the target traits are expressed before or after flowering, determining how efficiently unfavourable individuals can be removed to ensure that they are not passing on genetic material to the next generation. One of the most popular strategies for outcrossing crop species is hybrid breeding. Here, two genetically distant inbred lines are crossed to generate fully uniform F1 hybrids that show a significantly higher performance than both parents. This approach takes advantage of a phenomenon called heterosis (or hybrid vigour) and while different theories have been developed, its biological basis remains elusive. In maize, spectacular yield increases have been realised since the implementation of hybrid breeding in the early twentieth century. In rice there are hybrids that produce up to 30% more yield than common inbred lines. However, a major challenge is to practically ensure directed crossing and efficient production of hybrid seed. Breeders typically deploy genetic sterility mechanisms to make sure that genotype A is only pollinated with pollen from genotype B without pollen contamination from other sources (e.g. other genotypes or self-pollination), although chemical hybridization agents that are typically less efficient are used for some species. This restricts the availability of hybrid varieties for some crops, like wheat and barley. For commercial breeders hybrid varieties are very attractive because farmers cannot regrow the seeds they harvested but have to buy new seeds in every growing season. This is because seeds harvested from F1 hybrids (i.e. the F2 generation) will cause serious yield decreases (due to the 1:2:1 segregation), protecting IP of the hybrid variety and promoting higher profits from seed sales each year.

## Who is breeding the crop varieties?

Crop improvement programs are run in both the public and private sector. Public plant breeding programs typically produce germplasm, which is freely available to producers, researchers and other breeders, although there are IP regulations and material transfer agreements involved. On the other hand, seeds produced by private plant breeding programs underlie stricter IP regulations and have to be purchased through the breeding company or the contracted seed distributor. Several international research institutions run public crop breeding programs. For example, CGIAR represents a very large global partnership consisting of 15 agricultural research organisations whose joint agenda aims at improving global future food security, reducing poverty and improving human health and nutrition. Their joint investments into crop improvement run into the billions of US dollars. One of the partners is CIMMYT (The International Maize and Wheat Improvement Centre) based in Mexico, which is leading the wheat and maize improvement programs. CIMMYT has developed numerous varieties grown on millions of hectares worldwide. Another important public crop improvement organisation, which is heavily involved in the improvement of rice varieties, is the International Rice Research Institution (IRRI) based in the Philippines, representing the largest non-profit agricultural research organisation in Asia. Public crop improvement programs are also important for ‘pre-breeding’, which bridges discovery research and applied crop breeding. On the other hand is the private plant breeding sector, which is dominated by big multinational companies like Bayer, Syngenta and Corteva. These companies produce and commercialise seeds of highly productive varieties that can be purchased by farmers. In Europe, for example, there is a strong mid-tier for plant breeding consisting of small to mid-scale companies. While the big players mostly conduct breeding research using their own facilities and in-house resources, smaller companies typically depend on collaborative R&D activity with public research institutions and/or other small companies.

## What was the green revolution?

The Green Revolution describes the tremendous increase of grain yield associated with improved genetics and application of plant protection chemicals and mineral fertilizers. While it took almost 10,000 years for humans to produce one billion tons of grain globally, the Green Revolution led to a doubling of that amount in just 40 years between 1960 and 2000. A key driver of the Green Revolution was the introduction of so-called semi-dwarfing genes (reduced height, *Rht*-genes) in wheat. Varieties carrying the *Rht* genes were shorter (Fig. 1b) and much better at utilizing increased amounts of applied nitrogen. In comparison to taller varieties, which tend to lie flat on the ground (lodging) as a result of high nitrogen fertilization and/or increasing grain load, *Rht*-carrying varieties contribute to a better nitrogen use efficiency, preventing nitrogen from being wasted and polluting neighboring ecosystems [5]. Today, dwarfing genes are widespread in modern cereal varieties worldwide and a range of different *Rht* genes have been characterized. The first deployed *Rht* genes, including *Rht-B1b* (formerly *Rht1*) and *Rht-D1b 8b* (formerly *Rht2*) originated from the Japanese wheat variety ‘Norin 10’, which is a progeny of the Japanese semi-dwarf landrace ‘Daruma’ and an American high-yielding variety. Norin 10 was central for the creation of several important Green Revolution wheat varieties. Also for barley and maize the orthologous genes *sln1* and *dwarf8* were discovered. These genes generally encode transcription factors that target components of the gibberellin acid (GA) pathway, which regulate GA response. GA is a tetracyclic diterpenoid acid that is important for the onset of flowering and pollen development, as well as a key determinant of cell elongation and therefore plant height. Consequently, wheat and rice plants that carry semi-dwarfing genes are shorter and realize a higher harvest index, defined as the ratio of grain yield over the entire plant biomass. The advent of smaller, more stable varieties with a higher harvest index was accompanied by several positive effects, including an improved allocation of nutrients and assimilates to the grains and a reduction of residual plant biomass [5].

## How does climate change affect crop production?

Climate change is a generic term that describes the recent and forecasted change of multiple environmental parameters. Most of them, including atmospheric CO_2_ concentration, temperature and the frequency and amount of precipitation, affect plant growth. While a higher CO_2_ concentration usually increases photosynthesis, a lack of rainfall at critical developmental stages decreases crop yields. The record-breaking Millennium droughts in 1996/97, 2001–2003, 2006 and 2018 in Australia or 2003, 2010, 2015 and 2018 in Europe are examples for extreme effects of drought on crop production. On the other hand, like CO_2_ concentration, increasing temperature can accelerate plant growth due to a higher enzymatic activity. Beyond the temperature optimum, which is very crop and variety specific, higher temperatures result in heat stress, which is considered a major cause of wheat yield loss in developing countries. It has been estimated that each °C increase leads to a decrease of global wheat production of 6% [6]. Increasing temperature can also indirectly affect crop yields due to an increased occurrence of pests and diseases. Clearly, the magnitude of impact of climate change on crop yields will depend on the geographic region. For example, an increase in temperature of 4 °C has been forecasted to reduce wheat yields by 20–30% in tropical regions, whereas in temperate regions, the same temperature change will not likely lead to dramatic yield losses [7]. It is very difficult to attribute specific weather events to climate change since effects are mainly measurable as long-term trends. Furthermore, environmental effects on crop yields can vary strongly from year to year. There are arguments that with increased temperatures the atmosphere can hold more water. However, different spatiotemporal evaporation rates which are not synchronized with increased atmospheric water holding capacity drive changes in global precipitation. Hence, drought events are predicted to become more frequent and severe in many crop-growing regions. Plant breeding is expected to play a central role in meeting the challenge to adapt crops to future growth conditions.

## What is the genomics era of crop improvement?

Over the past ~ 10,000 years, crops were mainly improved through selection of superior individuals that showed characteristics favourable for human nutrition and production, but without enforced crossing involved. The formulation of Mendelian laws heralded the beginning of modern plant breeding, which has changed tremendously over the past 150 years. The introduction and continuous development of theoretical frameworks, including quantitative genetics principles and the rapid advances in the field of modern biotechnology and genomics, have made plant breeding a very complex discipline [2]. Modern plant breeding programs involve expert teams that combine very broad and different skillsets, such as genetics, statistics, agronomy, biochemistry, physiology, bioinformatics, molecular biology and economics, making them highly interdisciplinary. Advances in DNA sequencing technologies have revolutionised crop breeding and research, opening up the ‘genomics era’ of crop improvement. Today, whole-genome reference DNA sequences are available for most important crop species and very cost-efficient genotyping platforms to ‘DNA fingerprint’ plants have been developed. The DNA marker of choice is typically single-nucleotide-polymorphism (SNP) markers because they are ubiquitous in crop genomes and very easy and cost-efficient to score. It has therefore become standard practice in modern crop improvement to genotype large populations of plants with thousands to tens of thousands of markers on a routine basis. Even whole-genome resequencing data are becoming increasingly available, providing unprecedented insights into structural diversity across crop genomes [8]. Using the latest statistical genetics approaches, vast amounts of genotype data are used for various purposes. For instance, a very promising modern selection strategy that incorporates genome-wide DNA marker information is ‘genomic selection’ in which statistical models or machine learning algorithms are deployed to link genomic polymorphisms to phenotypic variation. The underlying theoretical foundation of the approach is that genes (or more generic quantitative trait loci (QTL)) that affect the trait of interest (e.g. grain yield) are tagged by DNA markers, allowing one to derive effect approximations for each of those QTL on the target trait. A prediction equation is used to calculate a genomic estimated breeding value for each genotype, based on whole-genome marker profiles, without actually testing those genotypes in field trials. This allows breeders to predict genotype performance as soon as DNA marker profiles can be generated (i.e. at seedling stage). Ultimately, the time until selection decisions are being made is significantly decreased, which leads to increased genetic gain per unit of time. Since its formal introduction in 2001, genomic selection has led to tremendous increases in genetic gain in animal breeding (e.g. dairy cattle) and it has a huge potential for crop improvement as well.

## Can new technologies speed up crop improvement?

The rate of improvement of genetic yield potential has to be increased beyond the rates currently achieved in ongoing breeding programs to protect global food security in times of rapid population growth and climate change. Thus, new or different approaches are needed to accelerate the crop breeding process. Over the past decades, numerous technologies have emerged that can accelerate plant breeding, such as genomic selection (described above). In addition to genomics approaches described above, other new methodologies such as gene editing technology are fast-evolving and protocols have been refined for most major crop species. In CRISPR gene editing systems, guide RNA directs the Cas9 enzyme to the target DNA site and cuts the DNA. This can be used to activate or deactivate alleles of a target gene to enhance plant performance, e.g. through improving disease resistance or drought tolerance [9]. Despite the promise of gene editing and strong support from the scientific literature regarding safety and sustainability, many countries have employed strict legal frameworks as a consequence of controversial discussions—mainly ideology-driven—and a rejection of genetically modified food. On the other hand, a very widely used and accepted breeding method is mutation breeding, which uses chemicals or radiation to induce random mutations throughout the genome instead of genetically engineered (targeted) mutation. In fact, spontaneous mutations in the plant genome occur naturally. For example, in a wheat field the size of one hectare, about 20 billion mutations occur each year (Prof. Detlef Weigel, personal communication). This is why the majority of the plant science community argue that mutations induced using genome editing where no foreign DNA is introduced should be considered a non-GM tool. Alternatively, ‘speed breeding’ developed by Dr. Lee Hickey and colleagues provides a non-GM route to rapidly introduce or stack new trait variation. This technique uses controlled environmental conditions and extended photoperiods to achieve up to six generations per year, instead of just one or two in the field. This can speed up the development of inbred lines following a cross, similar to doubled haploid technology, which is a lab-based technique that has been a revelation for breeding crops like maize and winter wheat. Most of the modern technologies have been proven to assist the development of improved crop varieties. However, more efficient breeding strategies that effectively combine these technologies could lead to a step-change in the rate of genetic gain. Ongoing investment from the public and private sectors is necessary to build and maintain capacity for sustained crop improvement to ensure the development of crops that are capable of feeding the world in the future.
